# Stressor control and regional inflammatory responses in the brain: regulation by the basolateral amygdala

**DOI:** 10.1186/s12974-023-02813-x

**Published:** 2023-05-27

**Authors:** Austin M. Adkins, Emily M. Colby, Woong-Ki Kim, Laurie L. Wellman, Larry D. Sanford

**Affiliations:** 1 Sleep Research Laboratory, Eastern Virginia Medical School, P.O. Box 1980, Norfolk, VA 23507 USA; 2 Center for Integrative Neuroscience and Inflammatory Diseases, Eastern Virginia Medical School, P.O. Box 1980, Norfolk, VA 23507 USA; 3 Pathology and Anatomy, Eastern Virginia Medical School, P.O. Box 1980, Norfolk, VA 23507 USA; 4 Microbiology and Molecular Cell Biology, Eastern Virginia Medical School, P.O. Box 1980, VA 23507 Norfolk, USA

**Keywords:** Stressor controllability, Amygdala, Optogenetics, Parainflammation, Nanostring

## Abstract

Increasing evidence has connected the development of certain neuropsychiatric disorders, as well as neurodegenerative diseases, to stress-induced dysregulation of the immune system. We have shown that escapable (ES) and inescapable (IS) footshock stress, and memories associated with ES or IS, can differentially alter inflammatory-related gene expression in brain in a region dependent manner. We have also demonstrated that the basolateral amygdala (BLA) regulates stress- and fear memory-induced alterations in sleep, and that differential sleep and immune responses in the brain to ES and IS appear to be integrated during fear conditioning and then reproduced by fear memory recall. In this study, we investigated the role of BLA in influencing regional inflammatory responses within the hippocampus (HPC) and medial prefrontal cortex (mPFC) by optogenetically stimulating or inhibiting BLA in male C57BL/6 mice during footshock stress in our yoked shuttlebox paradigm based on ES and IS. Then, mice were immediately euthanized and RNA extracted from brain regions of interest and loaded into NanoString® Mouse Neuroinflammation Panels for compilation of gene expression profiles. Results showed differential regional effects in gene expression and activated pathways involved in inflammatory-related signaling following ES and IS, and these differences were altered depending on amygdalar excitation or inhibition. These findings demonstrate that the stress-induced immune response, or “parainflammation”, is affected by stressor controllability and that BLA influences regional parainflammation to ES or IS in HPC and mPFC. The study illustrates how stress-induced parainflammation can be regulated at the neurocircuit level and suggests that this approach can be useful for uncovering circuit and immune interactions in mediating differential stress outcomes.

**Supplementary Information:**

The online version contains supplementary material available at 10.1186/s12974-023-02813-x.

## Introduction

Stress-related dysregulation of the immune system is increasingly being linked to health risks [1, 2], and to increased inflammation [3–5], which is implicated in neurodegenerative diseases [6–13] and neuropsychiatric disorders [7, 14–17]. However, stressors are most often encountered without producing pathological changes. The difference between successful and unsuccessful coping with stress may involve characteristics of the stressor (e.g., controllability, intensity, duration) and the resilience or vulnerability of the organism undergoing stress [18, 19]. How these factors alter the impact of stress on the immune system is poorly understood.

Research on stressor controllability [20, 21] suggests that the outcome of a challenging event involves organismic processes beyond the simple elicitation of a stress response. Consistent with this view, we have found that controllable stress (escapable shock training [ES] and presentation of contextual reminders of ES) and yoked uncontrollable stress (inescapable shock training [IS] and presentation of contextual reminders of IS) can produce significant differences in several behavioral and neurobiological responses despite virtually identical activation of the hypothalamo–pituitary–adrenal (HPA) axis, similar stress-induced hyperthermia (SIH), and the same overt fear behavior [22–24]. These alterations include directionally different changes in post-stress sleep [25, 26] and differential activation in stress-regulatory regions in the brain [27, 28]. We have also found that ES, and fear memories associated with ES, promote a down-regulation of many genes linked to pro-inflammation and an up-regulation of genes linked to neuroprotection whereas IS, and fear memories associated with IS, promote a down-regulation of genes linked to neuronal protection, but an up-regulation of genes involved in pro-inflammatory pathways [29]. These findings suggest that controllable and uncontrollable stress can produce significant regulatory differences in inflammatory-related pathways despite virtually identical peripheral stress responses.

The brain is responsive to immune challenges, and these responses can also be regionally distinct. For example, shortly after peripheral administration of bacterial lipopolysaccharide (LPS), the amygdala becomes activated and shows local synthesis of pro-inflammatory cytokines [30, 31]. Both LPS and IL-1β increased spontaneous neuronal firing in the basolateral nucleus of the amygdala (BLA) within 30 min of peripheral administration [32]. The ventral, medial and dorsomedial prefrontal cortex (PFC) also respond to peripheral inflammation, as indicated by increased functional magnetic resonance imaging activity [33, 34]. The BLA, PFC, hippocampus (HPC), and hypothalamus have established roles in responding to, and regulating, responses to stress [21, 35–38]. Stress-induced activation in these regions is associated with many hallmarks of neuroinflammation, including microglia stimulation and increased neuroinflammatory signaling [16]. However, stress-induced changes in neuroimmune functionality occur in the absence of disease, infection, or injury. An alternative term for neurobiological adaptations to stress associated with an immune state, “parainflammation”, has recently been coined [39]. How stress-induced parainflammation responses are regulated at the neurocircuit level is not known. In this study, we determined whether the differential parainflammation responses within HPC and the medial PFC (mPFC) associated with controllable and uncontrollable stress, modeled in our yoked ES and IS paradigm, are regulated by the BLA using optogenetics to selectively activate or inhibit BLA glutamatergic neurons during stress.

## Materials and methods

### Subjects

Male, C57BL/6 mice, 8–9 weeks of age and 20–25 g on arrival, were obtained from Charles River Laboratories (Wilmington, MA, USA) and individually housed for the duration of the study. Food and water were available ad libitum. Housing rooms were kept on a 12:12 light:dark cycle and ambient temperature was maintained at 24.5 °C ± 0.5 °C. All procedures were conducted in accordance with the National Institutes of Health Guide for the Care and Use of Experimental Animals and were approved by Eastern Virginia Medical School’s Institutional Animal Care and Use Committee (Protocol#: 17-015).

Mice were randomly divided into three ES treatment groups (ES, ES with excitatory light (ESOe) or ES with inhibitory light (ESOi)), three IS treatment groups (IS, ISOe and ISOi), and two home cage (HC or HC with eYFP control construct (CHCO)) control groups (*n* = 4–6 per group). Previously, we have reported stress-related measures, such as sleep or inflammatory-related gene expression, does not differ greatly between HC and a control group which was placed in the shuttlebox chamber on each experimental day but not shocked [mock trained (MT)] [29]. Yet, how optogenetic inhibition or excitation may alter inflammatory-related gene expression in this control group is not known. Therefore, in this current study, we also included two MT control groups [MT with excitatory vector/light (CMTOe) or MT with inhibitory vector/light (CMTOi)].

### Virus vector construct

Purified adeno-associated virus preparations (AAV5) containing either-CaMKIIα-eNpHR3.0-eYFP-WPRE (NpHR) for inhibition or containing CaMKIIα-hChR2(H134R)-eYFP (hChR2) for activation. A CaMKIIα-eYFP (eYFP) construct was used as a control. All viral constructs were obtained from the UNC Virus Vector Core Facility (University of North Carolina at Chapel Hill). CaMKII enables selective targeting of a subpopulation of glutamatergic neurons and the construct has been used in studies examining the role of the amygdala in the regulation of anxiety behaviors [40] and fear learning [41] as well as in studies of the influence of BLA on other brain regions [42]. The final viral concentration for of each construct injection was 1.5 × 10^12^ virus molecules/ml in 350 mM NaCl, 5% d-Sorbitol.

### Surgery

All surgical procedures were conducted during the light period with the mice under isoflurane anesthesia as inhalant (5% induction; 2% maintenance). All ESOi/e, ISOi/e, CMTOi/e, and CHCO animals received prophylactic potassium penicillin (0.08 mg/g), gentamicin (0.005 mg/g) and dexamethasone (0.005 mg/g) subcutaneously. Microinjection cannulae (26-guage) connected to a syringe pump (BSP-99M, Braintree Scientific Inc., Braintree, MA, USA) were stereotaxically placed bilaterally above BLA (− 1.5 mm AP,  ± 2.9 mm ML, − 4.7 mm DV) for administration of constructs containing either NpHR or hChR2, or eYFP only. Each injection delivered 0.5 µl of virus vector at a flow rate of 0.1 µl/min. Cannulae remained in place for an additional 5–10 min to allow diffusion of viral particles away from the injection site. Custom-made optic probes (200 µm, conical tip, mated to metal ferules) were then implanted directly above the injection sites and secured to the skull using dental cement. Ibuprofen (30 mg/kg, oral) was continuously available in each animal’s drinking water for 24–48 h preoperatively and for a minimum of 72 h post-operatively to alleviate potential operative pain. The animals were given at least four weeks for recovery and to allow viral transduction. During that period, the animals were kept undisturbed except for post-surgical monitoring and weekly bedding changes.

### Training procedures

On experimental days 1 and 2, animals were shock trained (ST) with ES or IS (20 footshocks, 0.5 mA, 5.0 s max. duration, 1 min inter-trial intervals) in a shuttlebox (Coulbourn Instruments, Model E10-15SC). Shock presentation began 5 min after the mice were placed in the shuttleboxes and was controlled by a Coulbourn Graphic State V2.1 software via Coulbourn Precision Regulated Animal Shockers (Model E13-14). The ES groups had the ability to learn they could behaviorally terminate the footshock by moving to the opposite shuttlebox chamber; the yoked IS groups could not control the shock. Termination of shock for an ES mouse also terminated the shock to its yoked IS mouse in a separate shock chamber (Coulbourn Instruments, Model E10-15SC), ensuring each yoked set of mice received the same duration of shock. Thus, a pair of mice received identical shock, but it was characterized as either controllable or uncontrollable based on ability to escape. Mice were undisturbed during 5-min pre- and post-ST periods. All training took place during the light period in the 4th hour of the light period.

For optogenetic manipulation of BLA, mice were connected bilaterally to optic fibers, and placed in a shock chamber (Coulbourn Instruments) for ST as described above. Doric Lenses, Inc. Hybrid MultiLED Driver Software (Version 2.4.3) was used to control the timing and duration of light. Excitatory (ESOe/ISOe, 470 nm (blue), 20 ms pulses at 20 Hz presented at 5 s intervals) or inhibitory (ESOi/ISOi, 590 nm (amber), constant) light was produced via LEDs (Model: LEDFRJ-B/G_FC; Doric Lenses, Inc.). Light output was measured by an optical power meter and adjusted to ~ 10 mW at the optic fiber tip. Procedures started during the 4th hour of the light period. Light stimulation to activate hChR2 occurred at the start of shock presentations during ST and continued until the last shock presentation. Light stimulation to activate NpHR started prior to ST and continued for the duration of training. Mice received two identical consecutive days of ST. HC and CHCO groups did not experience the shuttlebox or shock, nor did the CHCO group experience the hookup to optic fibers. The MT control groups microinjected with either NpHR (CMTOi) or hChR2 (CMTOe) were connected bilaterally to optic fibers, and placed in the shock chamber on each experimental day but not shocked. CMTOi and CMTOe groups received the inhibitory or excitatory light stimulation as described above.

### RNA extraction

All groups were euthanized immediately after training on the second ST day via isoflurane sedation (inhalant: 5%, ≤ 5 min duration) and perfused with PBS. Brains were extracted and regions of interest (whole HPC and mPFC) micro-dissected, snap frozen and stored in RNAlater (ThermoFisher Scientific) at − 80 °C until analysis. RNA was isolated using the Qiagen RNeasy Mini Kit. Samples from each region were loaded into NanoString^®^ Mouse Neuroinflammation Panels. Results from the panels were uploaded to the nSolver database (Version 4.0.70; NanoString Technologies; Seattle, WA, USA) to assess relative levels of inflammatory markers within the panels. Gene expression and pathway profiles were compiled for each group to assess expression levels relative to the stress response following ST.

### Statistical analyses

Data were normalized to Nanostring’s internal positive and negative controls to account for slight differences in assay efficiencies. The normalized gene counts for each gene in each assay were then divided by the appropriate normalization factor and averaged for the samples of each mRNA type to generate counts normalized to the internal reference genes. Fold changes in gene transcript levels were determined relative to basal levels detected in the HC (or CHCO) group. Relative fold changes in transcript levels for each determined gene were compared between groups. The data were analyzed within nSolver using multiple *t*-tests with Benjamini–Yekutieli correction to control for the false discovery rate (FDR). Further two-way analyses of variance (ANOVA) with Tukey’s post hoc analysis guided by the ANOVA (alpha = 0.05) on group and related gene expression was performed where appropriate. The following comparisons were evaluated: CMTOi compared to CHCO, CMTOe compared to CHCO, CMTOi versus CMTOe, ES compared to HC, IS compared to HC, ES versus IS, ESOi compared to CHCO, ISOi compared to CHCO, ESOi versus ISOi, ESOe compared to CHCO, ISOe compared to CHCO, ESOe versus ISOe.

## Results

### Signaling pathways related to inflammation

Pathway regulation scores were determined using the nSolver database via directed global significance scores of overlaid differential gene expression data for sets of genes grouped by biological function relative to HC (or CHCO). This analysis measures the extent to which genes within a given set are up- or down-regulated with the independent variable.

When examining inflammatory-related pathway scores in the CMTO group compared to CHCO, minimal differences occurred (Additional file 1: Fig. S1A–D). In HPC, regardless of excitatory or inhibitory optogenetic manipulations, many pathways were not differentially regulated (no more than 0.5–onefold change up or down) in the CMTO group relative to CHCO (Additional file 1: Fig. S1A, C). In mPFC, regardless of excitatory or inhibitory optogenetic manipulations, many pathways were generally unchanged, or suppressed, compared to CHCO (Additional file 1: Fig. S1B, D). Due to the qualitative differences in responses between animals that received footshock and those that did not, we focused on comparisons between ES and IS (and ESOi/e and ISOi/e) groups that experienced footshock in our stressor controllability paradigm.

#### Stressor controllability

##### No amygdalar manipulation

**Fig. 1:**
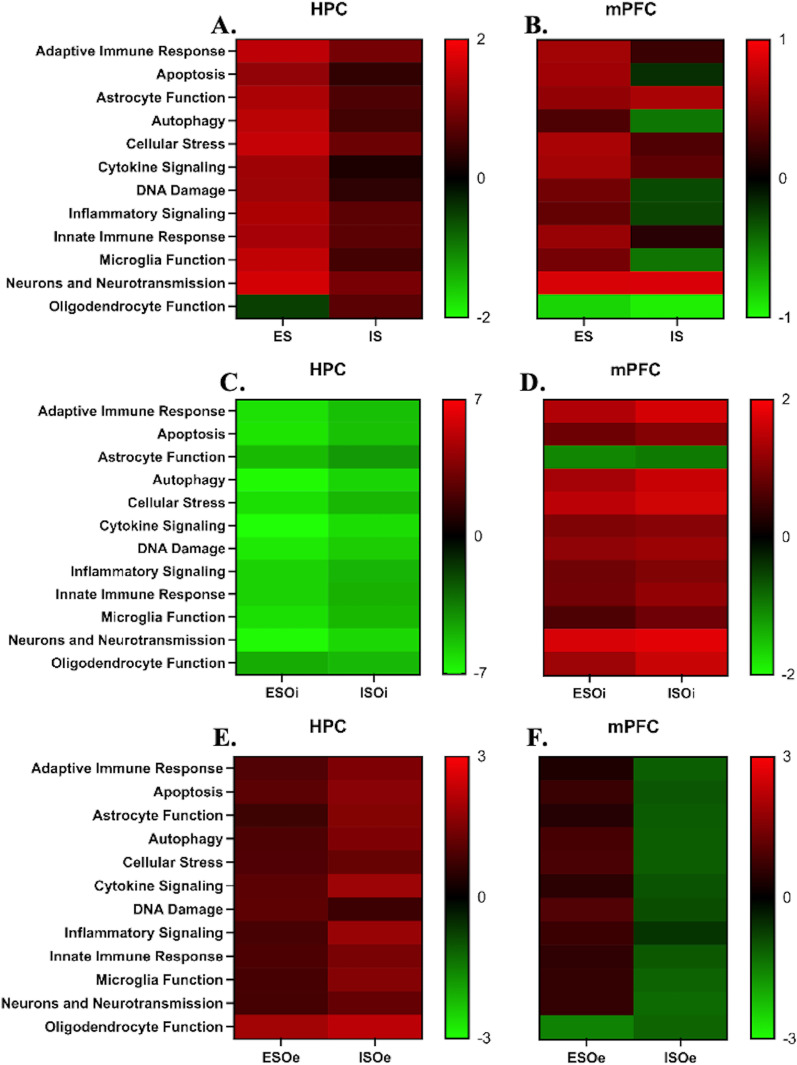
Changes to BLA input during stressor controllability differentially activates immune-related pathways in a regional manner. Heatmap displaying sample’s directed global significance pathway scores for hippocampus (HPC) and medial prefrontal cortex (mPFC) in **A**, **B** escapable (ES) and inescapable (IS) stress groups compared to home cage (HC); **C**, **D** ES and IS groups with inhibitory opto (ESOi and ISOi, respectively) compared to home cage with control vector (CHCO), and **E**, **F** ES and IS groups with excitatory opto (ESOe and ISOe, respectively) compared to CHCO. Red denotes neuroinflammatory-related pathway gene sets whose genes within exhibit extensive over-expression; green denotes gene sets with extensive under-expression. Mean scores are plotted to show how they vary across treatment conditions

In HPC, the ES and IS groups displayed distinct inflammatory-related pathway profiles compared to HC (Fig. 1A). The ES group exhibited greater up-regulation of these pathways compared to the IS group except in pathways related to oligodendrocyte function, where there was a greater down-regulation. The IS group also displayed up-regulation of pathways relative to the HC group, but they were less than observed in the ES group. In mPFC, the ES and IS groups displayed distinct inflammatory-related pathway profiles (Fig. 1B). The ES group exhibited greater up-regulation of these pathways compared to the IS group except in pathways related to oligodendrocyte function, where there was a greater down-regulation. The IS group displayed substantial down-regulations of all pathways except in those related to astrocyte function, cellular stress, cytokine signaling, and neurons and neurotransmission.

##### Amygdalar inhibition

In HPC, the ESOi and ISOi groups exhibited a dramatic suppression of all inflammatory-related pathway profiles compared to CHCO (Fig. 1C). In mPFC, ESOi and ISOi groups displayed an enhancement of all inflammatory-related pathway profiles with the exception of pathways related to astrocyte function (Fig. 1D).

##### Amygdalar activation

In HPC, the ESOe and ISOe groups showed an enrichment of all inflammatory-related pathway profiles compared to CHCO, and this on average was higher in the ISOe group compared to the ESOe group (Fig. 1E). In mPFC, the ESOe group also showed an enhancement of all inflammatory-related pathway profiles except for pathways related to oligodendrocyte function. The ISOe group displayed a suppression of all pathway regulatory profiles (Fig. 1F).

### Gene expression related to inflammation

Genes were tested for differential expression within nSolver in response to each covariate using a single linear regression fit for each individual gene to predict expression and grouped by biological pathway. Data were analyzed within nSolver as discussed above.

When examining inflammatory-related gene expression via relative mRNA levels in the CMTO group compared to CHCO, minimal differences were observed in either HPC or mPFC regardless of excitatory or inhibitory optogenetic manipulations (Additional file 1: Fig. S2A–D). In general, in both HPC and mPFC CMTOi/e groups exhibited a slight up-regulation of genes related to neurotransmission (*Homer1*; *Dlx1*; *Grm3*; and *Gria4*, *p* < 0.05 or less for all comparisons). CMTOi/e groups also exhibited an up-regulation of select genes associated with inflammatory signaling (e.g., *CD40*; *Tnfrsf25*; *Egr1; and Socs3*, *p* < 0.05 or less for all comparisons). Compared to CHCO, CMTOi/e groups also exhibited a down-regulation of genes related the immune response (e.g., *C1qb*; *C1qc*; and *Traf6*, *p* < 0.05 or less for all comparisons), immune cell activation, signaling, and recruitment (e.g., *Stgal6*; *Npnt*; and *Man2b1*, *p* < 0.05 or less for all comparisons), genes related to DNA damage (e.g., *Bax*; *Ccng2*; and *Rad1*, *p* < 0.05 or less for all comparisons), cellular stress (*Srxn1*; *Sod2*; and *Atg3*, *p* < 0.05 or less for all comparisons), and cell cycle-induced death (e.g., *Lig1*; *Cdc25a*; and *Nbn*, *p* < 0.05 or less for all comparisons), and genes related to repair mechanisms in response to damage such as: the clearance of dead cells and debris (*Mertk* and *Atg14*, *p* < 0.05 or less for all comparisons), matrix remodeling (*Pecam1*; *Ctss*; and *Esam*, *p* < 0.05 or less for all comparisons), and growth factor signaling (*Mef2c* and *Itgb5*, *p* < 0.05 or less for all comparisons) (Additional file 1: Fig. S2A–D). Again, due to the qualitative differences in the types of genes that were altered, we focused on comparisons between ES and IS (and ESOi/e and ISOi/e) groups that experienced footshock in our stressor controllability paradigm.

#### Stressor controllability

##### No amygdalar manipulation

**Fig. 2:**
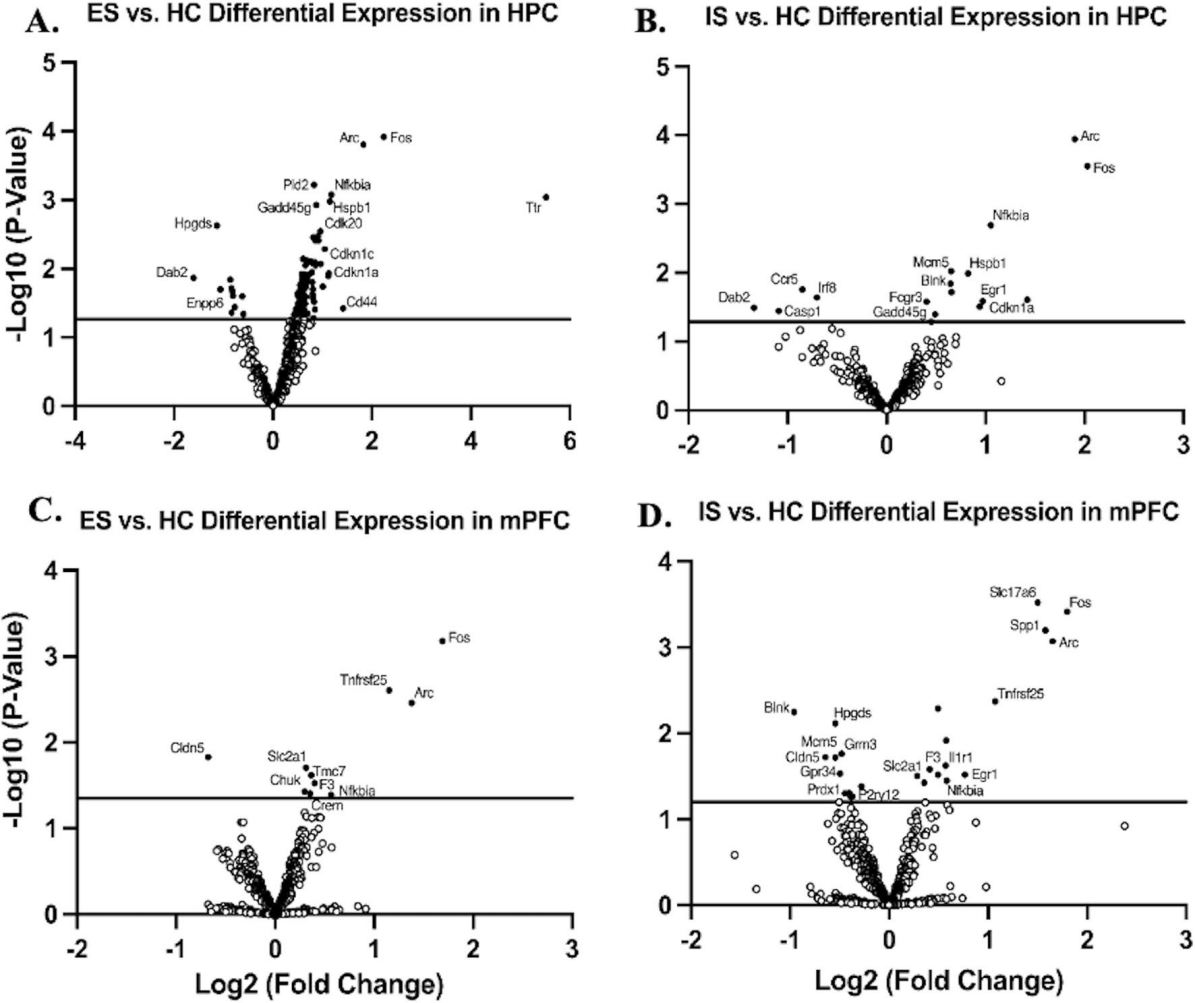
Controllability differentially regulates stress-induced inflammatory gene expression in a regional manner. Volcano plot displaying each gene expression levels in hippocampus (HPC) compared to home cage (HC) control following ST for **A** escapable (ES) and **B** inescapable (IS) stress groups and in medial prefrontal cortex (mPFC) for **C** ES and **D** IS groups. *p*-values were − Log10 transformed; statistically significant genes (*p* < 0.05) fall above the horizontal line. Highly differentially expressed genes fall to either side of the zero on the *x*-axis. The most relevant genes are labeled in the plot

Gene expression via relative mRNA levels within HPC revealed significantly different expression levels between ES and IS groups when compared to HC. ES mice showed a down-regulation of many genes associated with re-myelination and oligodendrocyte differentiation (*Enpp6* *p* < 0.05 and *Plp1*, *p* < 0.05); immune cell activation, signaling, and recruitment (*Ccr5*, *p* < 0.05; *Hpgds*, *p* < 0.01); and pro-inflammation (*Tlr4*, *p* = 0.01) (Fig. 2A). ES mice also showed an up-regulation of genes associated with the clearance of dead cells and debris (*Reln*, *p* < 0.01), proper protein folding (*Hspb1*, *p* = 0.001 and *Ttr*, *p* < 0.001), the regulation of cell cycle processes in response to stress (*Fos*, *p* = 0.0001; *Pld2*, *p* < 0.001; *Nfkbia*, *p* < 0.001; *Gadd45g*, *p* = 0.001; *Cdk20*, *p* < 0.01; *Cdkn1a*, *p* = 0.01), and proper signal transduction and neurotransmission (*Arc*, p = 0.0001; *Cdkn1c*, *p* < 0.01; *Cd44*, *p* < 0.05) (Fig. 2A). Compared to HC, IS showed a down-regulation of genes associated with immune cell activation (*Ccr5*, *p* = 0.01; *Irf8*, *p* < 0.05), pro-inflammation (*Casp1*, *p* < 0.05), and signal transduction and neurotransmission (*Dab2*, *p* < 0.05) (Fig. 2B). IS mice also showed an up-regulation of other genes involved in immune cell activation (*Blnk*, *p* = 0.01 and *Fcgr3*, *p* < 0.05), as well as genes involved in the regulation of cell cycle processes in response to stress (*Fos*, *p* < 0.001; *Nfkbia*, *p* < 0.01; *Mcm5*, *p* < 0.01; *Egr1*, *p* < 0.05; *Cdkn1a*, *p* < 0.05; *Gadd45g*, *p* = 0.05), proper protein folding (*Hspb1*, *p* = 0.01), and the *Arc* gene (*p* = 0.0001) (Fig. 2B).

Gene expression via relative mRNA levels within mPFC revealed significantly different expression levels between ES and IS groups when compared to HC. ES mice showed a down-regulation of *Cldn5* (*p* = 0.01), a gene associated with cell debris clearance and repair (Fig. 2C). ES mice also showed an up-regulation of genes associated with the regulation of cell cycle processes in response to stress (*Fos*, *p* < 0.001; *Tnfrsf25*, *p* < 0.01; *Nfkbia*, *p* < 0.05; *Chuk*, *p* < 0.05), and proper signal transduction and neurotransmission (*Arc*, *p* < 0.05; *Slc2a1*, *p* = 0.01; *Tmc7*, *p* < 0.05; *F3*, *p* < 0.05; *Crem*, *p* < 0.05) (Fig. 2C). Compared to HC, IS showed a down-regulation of genes associated with cell debris clearance and repair (*Cldn5*, *p* = 0.01; *Prdx1*, *p* < 0.05), immune cell activation and recruitment (*Blnk*, *p* < 0.01; *Hpgds*, *p* < 0.01; *P2ry12*, *p* < 0.05), cell cycle processes in response to stress (*Mcm5*, *p* = 0.01), and proper signal transduction and neurotransmission (*Grm3*, *p* = 0.01; *Gpr34*, *p* < 0.05) (Fig. 2D). IS also showed an up-regulation of other genes involved in cell cycle processes in response to stress (*Fos*, *p* < 0.001; *Tnfrs25*, *p* < 0.01; *Egr1*, *p* < 0.0302; *Nfkbia*, *p* < 0.05), pro-inflammation (*Spp1*, *p* < 0.001), signal transduction and neurotransmission (*Slc17a6*, *p* < 0.0001; *Arc*, *p* < 0.001; *F3*, *p* < 0.05; *Slc2a1*, *p* < 0.05), and immune cell activation and recruitment (*Il1r1*, *p* < 0.05) (Fig. 2D).

##### Amygdalar inhibition

**Fig. 3:**
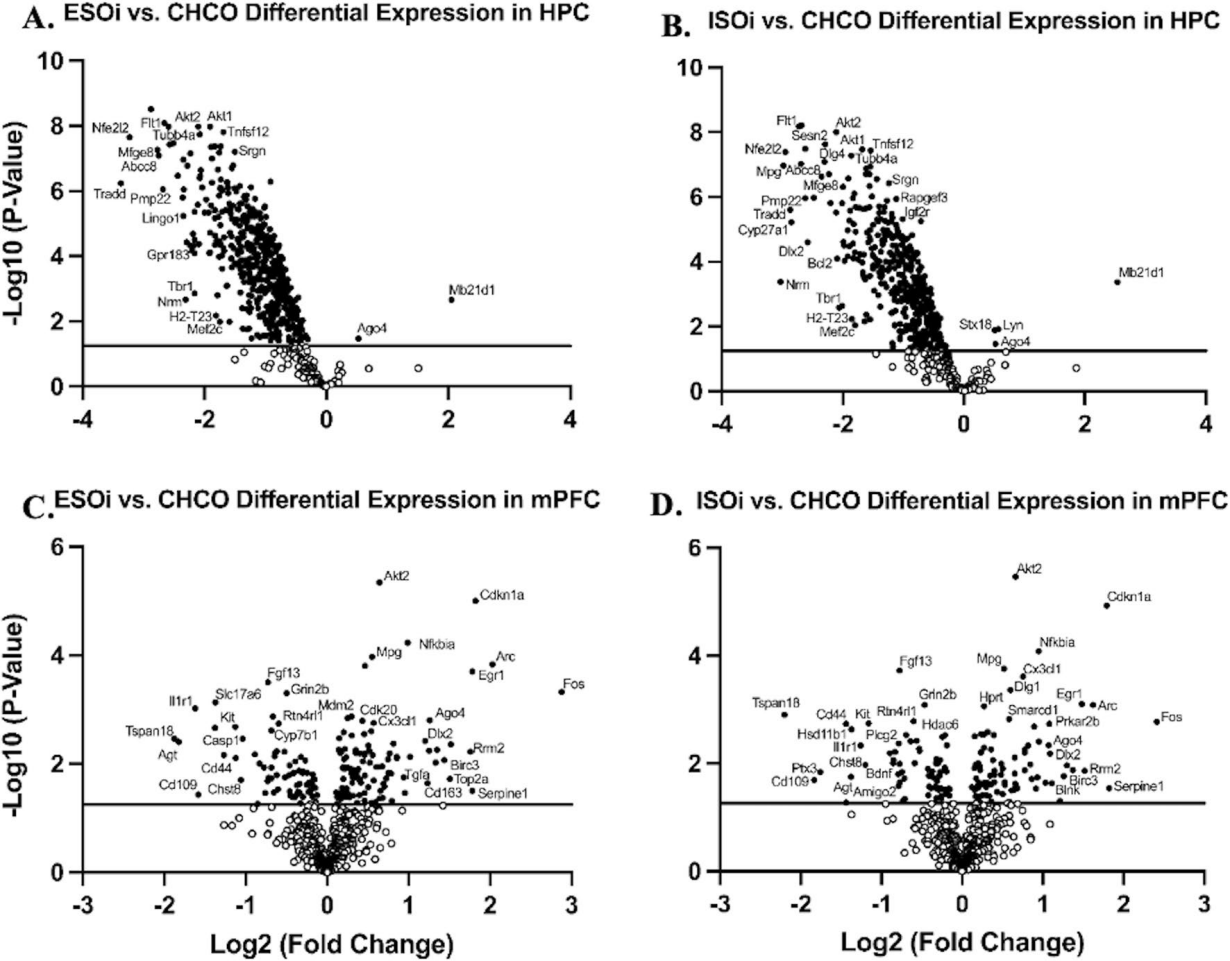
Inhibition of BLA eliminates differences in stressor controllability-induced inflammatory gene expression. Volcano plot displaying each gene expression levels in hippocampus (HPC) compared to home cage (CHCO) control following ST for **A** escapable stress with inhibitory opto (ESOi) and **B** inescapable stress with inhibitory opto (ISOi) groups and in medial prefrontal cortex (mPFC) for **C** ESOi and **D** ISOi groups. *p*-values were -Log10 transformed; statistically significant genes (*p* < 0.05) fall above the horizontal line. Highly differentially expressed genes fall to either side of the zero on the x-axis. The most relevant genes are labeled in the plot

Gene expression via relative mRNA levels within HPC revealed similar expression levels between ESOi and ISOi groups when compared to CHCO. ESOi mice showed a significant down-regulation in all gene expression, with the exception of one gene related to the adaptive immune response due to cellular stress (*Ago4*, *p* < 0.05) and one gene related to the innate immune response/inflammatory signaling (*Mb21d1*, *p* < 0.01) (Fig. 3A) which were significantly upregulated. ISOi mice also showed a significant down-regulation in all gene expression, with the exception of *Ago4* (*p* > 0.05) and *Mb21d1* (*p* > 0.01) as with the ESOi group, as well as one gene related to autophagy (*Stx18*, *p* = 0.01) and one gene related to the adaptive immune response (*Lyn*, *p* = 0.01) (Fig. 3B) which were significantly upregulated.

Gene expression assessed via relative mRNA levels within mPFC revealed similar expression levels between ESOi and ISOi groups when compared to CHCO. ESOi mice showed a down-regulation of multiple genes associated with inflammatory signaling (*Cyp7b1*, *p* < 0.01); microglia function (*Rtn4rl1*, *p* = 0.001; *Fgf13*, *p* < 0.001; *Chst8*, *p* = 0.02; *Tspan18*, *p* < 0.01); the adaptive immune response (*Grin2b*, *p* < 0.001; *Kit*, *p* < 0.01); neurons and neurotransmission (*Slc17a6*, *p* = 0.0001); apoptosis due to cellular stress (*Casp1*, *p* < 0.01; *Il1r1*, *p* = 0.001); and astrocyte function (*Cd44*, *p* < 0.01; *Cd109*, *p* < 0.05; *Agt*, *p* < 0.01) (Fig. 3C). ESOi mice also showed an up-regulation of other genes related to the adaptive immune response (*Akt2*, *p* < 0.00001; *Cdkn1a*, *p* < 0.00001; *Nfkbia*, *p* < 0.0001; *Mdm2*, *p* = 0.001; *Fos*, *p* < 0.001; *Ago4*, *p* = 0.001); cell cycle regulation (*Cdk20*, *p* = 0.001; *Rrm2*, *p* < 0.01); cytokine signaling (*Cx3cl1*, *p* = 0.001); DNA damage (*Mpg*, *p* = 0.0001); neurons and neurotransmission (*Arc*, *p* = 0.0001; *Dlx2*, *p* < 0.01; *Cd163*, *p* = 0.02); inflammatory signaling (*Egr1*, *p* < 0.001); apoptosis (*Birc3*, *p* < 0.01; *Top2a*, *p* < 0.05); astrocyte function (*Tgfa*, *p* < 0.05); and autophagy (*Serpine1*, *p* = 0.03) (Fig. 3C). Compared to CHCO, ISOi mice showed some similarities in the down-regulation of genes (as in the ESOi group) associated with microglia function (*Rtn4rl1*, *p* = 0.001; *Fgf13*, *p* = 0.0001; *Chst8*, *p* = 0.01; *Tspan18*, *p* = 0.001); the adaptive immune response (*Grin2b*, *p* < 0.001; *Plcg2*, *p* < 0.01; *Kit*, *p* = 0.001); neurons and neurotransmission (*Bdnf*, *p* < 0.01); apoptosis due to cellular stress (*Il1r1*, *p* < 0.01); cellular stress (Hda6c, *p* < 0.01); stress hormone regulation (Hsd11b1, *p* < 0.01); and astrocyte function (*Cd44*, *p* = 0.001; *Cd109*, *p* = 0.02; *Agt*, *p* < 0.05; *Amigo2*, *p* < 0.05; *Ptx3*, *p* = 0.01) (Fig. 3D). ISOi mice also showed a similar up-regulation of genes (as in the ESOi group) related to the adaptive immune response (*Akt2*, *p* < 0.00001; *Cdkn1a*, *p* = 0.000001; *Nfkbia*, *p* < 0.0001; *Fos*, *p* = 0.001; *Ago4*, *p* < 0.01; *Blnk*, *p* = 0.02); cell cycle regulation (*Rrm2*, *p* < 0.01); cytokine signaling (*Cx3cl1*, *p* = 0.0002); DNA damage (*Mpg*, *p* < 0.001); neurons and neurotransmission (*Arc*, *p* < 0.001; *Dlx2*, *p* < 0.01; *Dlg1*, *p* < 0.001); inflammatory signaling (*Egr1*, *p* < 0.001); apoptosis (*Birc3*, *p* = 0.01; *Hprt*, *p* < 0.001; *Prkar2b*, *p* = 0.001); transcriptional regulation (*Smarcd1*, *p* = 0.001); and autophagy (*Serpine1*, *p* = 0.02) (Fig. 3D).

##### Amygdalar activation

**Fig. 4:**
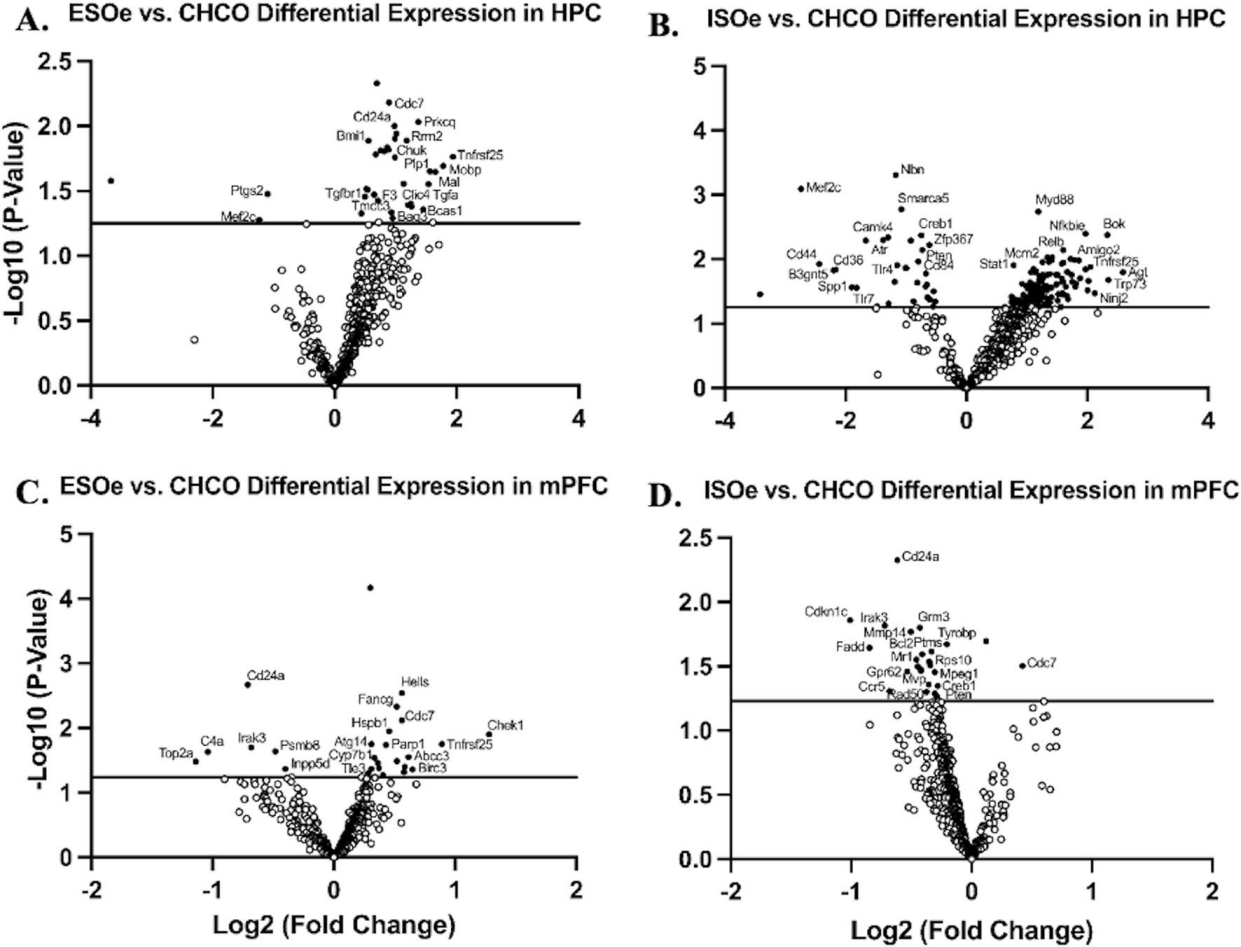
Activation of BLA amplifies differences in stressor controllability-induced inflammatory gene expression. Volcano plot displaying each gene expression levels in hippocampus (HPC) compared to home cage (CHCO) control following ST for **A** escapable stress with excitatory opto (ESOe) and **B** inescapable stress with excitatory opto (ISOe) groups and in medial prefrontal cortex (mPFC) for **C** ESOi and **D** ISOi groups. P-values were − Log10 transformed; statistically significant genes (*p* < 0.05) fall above the horizontal line. Highly differentially expressed genes fall to either side of the zero on the *x*-axis. The most relevant genes are labeled in the plot

Gene expression assessed via relative mRNA levels within HPC revealed distinctive expression levels between ESOe and ISOe groups when compared to CHCO. ESOe mice showed a significant down-regulation of two genes related to the innate immune response and microglia function (*Ptgs2*, *p* = 0.03; *Mef2c*, *p* = 0.05) (Fig. 4A). ESOe mice also showed an up-regulation in genes associated with cell cycle regulation (*Cdc7*, *p* < 0.01; *Rrm2*, *p* = 0.01); the adaptive immune response (*Prkcq*, *p* < 0.01; *Cd24a*, *p* = 0.01; *Chuk*, *p* = 0.01); protection from cellular stress (*Bmi1*, *p* = 0.01); oligodendrocyte function (*Plp1*, *p* = 0.02; *Mobp*, *p* = 0.02; *Mal*, *p* = 0.02; *Bcas1*, *p* < 0.05); apoptosis (*Tnfrsf25*, *p* = 0.01; *Bag3*, *p* < 0.05); astrocyte function (*Tgfa*, *p* = 0.02); autophagy (*Clic4*, *p* < 0.05); microglia function (*F3*, *p* < 0.05; *Tmcc3*, *p* < 0.05); and cytokine signaling (*Tgfbr1*, *p* < 0.05) (Fig. 4A). Compared to CHCO, ISOe mice showed a down-regulation of genes related to cell cycle regulation (*Nbn*, *p* < 0.001; *Smarca5*, *p* = 0.001; *Atr*, *p* < 0.01); growth factor signaling (*Mef2c*, *p* < 0.001; *Camk4*, *p* = 0.004; *Spp1*, *p* = 0.02); the adaptive immune response (*Creb1*, *p* = 0.004; *Pten*, *p* < 0.01; *Cd36*, *p* = 0.01); the innate immune response (*Tlr7*, *p* < 0.05); microglia function (*Zfp367*, *p* < 0.01); immune cell signaling (*Cd84*, *p* = 0.01); apoptosis (*Tlr4*, *p* = 0.01); and astrocyte function (*B3gnt5*, *p* < 0.01; *Cd44*, *p* = 0.01) (Fig. 4B). ISOe mice also showed a significant up-regulation of genes associated with apoptosis (*Myd88*, *p* = 0.001; *Bok*, *p* = 0.004; *Tnfrsf25*, *p* = 0.01); the adaptive immune response (*Nfkbie*, *p* = 0.003); cytokine signaling (*Relb*, *p* < 0.01; *Stat1*, *p* = 0.01); inflammatory signaling (*Mcm2*, *p* < 0.01); astrocyte function (*Amigo2*, *p* < 0.01; *Agt*, *p* = 0.01); DNA damage (*Trp73*, *p* = 0.02); and oligodendrocyte function (*Ninj2*, *p* = 0.03) (Fig. 4B).

Gene expression assessed via relative mRNA levels within mPFC revealed different expression levels between ESOe and ISOe groups when compared to CHCO. ESOe mice showed a down-regulation of genes associated with the adaptive immune response (*Cd24a*, *p* = 0.002; *Pmb8*, *p* = 0.02; *Inpp5d*, *p* < 0.05); apoptosis (*Irak3*, *p* = 0.02; *Top2a*, *p* = 0.03); and astrocyte function (*C4a*, *p* = 0.02) (Fig. 4C). ESOe mice also showed an up-regulation of genes related to apoptosis (*Hells*, *p* = 0.002; *Tnfrsf25*, *p* = 0.01; *Parp1*, *p* = 0.01; *Birc3*, *p* < 0.05); DNA damage (*Fancg*, *p* = 0.004); cell cycle regulation (*Cdc7*, *p* < 0.01; *Chek1*, *p* = 0.01); astrocyte function (*Hspb1*, *p* = 0.01); autophagy (*Atg14*, *p* = 0.01); microglia function (*Abcc3*, *p* = 0.03; *Tle3*, *p* = 0.05); and inflammatory signaling (*Cyp7b1*, *p* = 0.02) (Fig. 4C). Compared to CHCO, ISOe mice showed an up-regulation of one gene related to cell cycle regulation (*Cdc7*, *p* = 0.03) (Fig. 4D). ISOe mice also showed a significant down-regulation of genes associated with the adaptive immune response (*Cdk24a*, *p* < 0.01; *Tyrobp*, *p* = 0.02; *Creb1*, *p* = 0.04; *Pten*, *p* = 0.05); cell cycle regulation (*Cdkn1c*, *p* = 0.01; *Rad50*, *p* = 0.05); apoptosis (*Irak3*, *p* = 0.01; *Fadd*, *p* = 0.02; *Bcl2*, *p* = 0.02); astrocyte function (*Grm3*, *p* = 0.01); cellular stress (*Mmp14*, *p* = 0.01); microglia function (*Ptms*, *p* = 0.02; *Mr1*, *p* = 0.01; *Mvp*, *p* = 0.03; *Rps10*, *p* = 0.03); oligodendrocyte function (*Gpr62*, *p* = 0.03); cytokine signaling (*Ccr5*, *p* < 0.05); and inflammatory signaling (*Mpeg1*, *p* = 0.03) (Fig. 4D).

## Discussion

The effects of stress on the immune system are generally reported in the context of the effects of experimental paradigms (e.g., footshock, swim stress, restraint [43]; social defeat [44]) in which the subject is the passive recipient of a stressful manipulation. Effects are also often discussed in the context of a general effect of stress on the immune system [45–47]. However, our work [29, 48], and that of others [49], demonstrate that giving animals the ability to engage in behaviors that can modify stressor perception and/or stress outcomes can also alter stress-induced parainflammatory responses. The current study demonstrates that many of these responses can be regulated by the amygdala, as optogenetic inhibition or activation of BLA during stressor presentation altered immune pathway activation and gene expression related to inflammation, with regional differences between HPC and mPFC observed.

To determine whether optogenetic activation or inhibition of BLA also led to immune changes in HPC and mPFC irrespective of stress (footshock), we measured inflammatory-related gene expression in the MT control groups microinjected with the excitatory (CMTOe) or inhibitory (CMTOi) viral constructs. Overall, we found that CMTOi/e groups exhibited a low-grade inflammatory response in both HPC and mPFC as indicated by a slight up-regulation of a subset of genes related to inflammatory signaling relative to the CHCO group. This was likely induced by the handling of the animals necessary for tethering to the optic fiber cables required for the study and may have involved responses to the novel environment. Additionally, CMTOi/e animals showed an up-regulation of genes related to neurotransmission which might have also involved responses to the novel environment.

Importantly, these inflammatory genes in the CMTOi/e mice (e.g., *CD40*; *Tnfrsf25*; *Egr1; and Socs3*) were different compared to animals that experienced footshock (e.g., *Tlr4* for ES/IS, *Mb21d1* for ESOi/ISOi, and *Tgfbr1* and *Mcm2* for ESO/ISOe, respectively). Additionally, the inflammatory signaling in CMTOi/e groups were not coupled with an activation of the immune system or recruitment of immune cells, as indicated by a down-regulation of many genes associated with immune activation, signaling, and cell recruitment. There also was no indication of possible damage induced by inflammation or immune system activation that was seen in groups that experienced ES or IS, as many genes related to repair mechanisms that would be activated in response to damage were down-regulated in the CMTOi/e groups. Therefore, the general handling of the animal, and exposing the animal to a novel environment may evoke the system to respond by “priming” itself for a dangerous or stressful experience; however, when not coupled with an intense stressor (such as footshock), the response appears to subside.

Therefore, these findings suggest that optogenetic manipulation of BLA alone does not induce substantial alterations in immune responses within other regions of the stress-responsive circuit. Only when BLA manipulations were paired with footshock did significant differences in regional immune responses occur. Thus, we primarily focused on comparisons between ES and IS (and ESOi/e and ISOi/e) groups that experienced footshock in our stressor controllability paradigms.

### The stress-responsive circuit

BLA, HPC, and mPFC interact to form a three-way circuit involved in various aspects of the stress response [21, 35–38] and the formation of stress-related and fear memories [50, 51], and their interactions that are central to current concepts regarding stress-related psychopathology [52, 53]. The amygdala is important in the regulation of behavioral, physiological, and neuroendocrine responses to stress [54–56], and we have demonstrated that BLA regulates stress- and fear memory-induced alterations in sleep [57, 58]. The HPC forms contextual associations with fearful stimuli and takes part in the retrieval of fear memories [59–62], and determines if a fearful context is threatening or non-threatening [62, 63]. In particular, the ventral HPC and ventral subiculum play a role in modulating HPA-regulated stress responses [64]. The mPFC has a demonstrated role in the perception of control and in mediating the consequences of stress [21, 38]. For example, blocking activation of the ventromedial PFC with muscimol in rats presented with ES produced failure in escape learning and greater fear conditioning [38]. By comparison, activation of ventromedial PFC with picrotoxin prior to IS promoted subsequent escape learning in rats provided with an opportunity to escape shock in a shuttlebox [38]. Interestingly, we previously demonstrated that peri-shock BLA inhibition during IS led to c-Fos activation in mPFC and differential activation of brainstem (dorsal raphe nucleus and locus coeruleus) targets [65] consistent with those seen with mPFC activation [66, 67], thereby suggesting BLA input into the neurocircuit that evaluates stressor controllability. The BLA does not affect basal HPA activity or HPA axis responses to social interaction, novelty, restraint, ether, or cold [68], and we have seen no indication that it alters the stress response (as indicated by SIH) to IS [57, 58]. These findings indicate that BLA is not necessary for normal expression of acute HPA axis responses; however, the current results demonstrate that it can play a significant role in regulating stress-induced immune responses in the brain, and that its influence may vary by region.

### Stressor controllability

In the current study, we found that both ES and IS alone can induce distinct regional immune responses that are observable immediately following stress exposure. Overall, ES resulted in a general suppression of inflammatory signaling and increased neuroprotection whereas IS resulted in a dysregulation of neuroprotection and increased activation of pro-inflammatory signaling. The differences in gene expression elicited by ES and IS were also distinct in HPC and mPFC, suggesting that regions connected at the circuit level can have immune responses that are locally unique, most likely due to their roles at the individual level in the greater stress-responsive circuit. These findings complement our previous published results [27–29, 48].

Observations in inflammatory-related gene expression immediately following stress exposure in this study show similar up-regulation patterns of particular genes between ES and IS groups, and across regions. For example, *Hspb1*, *Fos*, *Nfkbia*, and *Arc* are all similarly upregulated following ES and IS exposure in both HPC and mPFC. We have also found that the expression of these four genes can also be elicited by fear memories of ES and IS, and their transcription changed 2 h following the stress response compared to immediately after [29]. For example, the expression of *Hspb1* remains similarly upregulated in both ES and IS; *Fos* and *Nfkbia* expression subsided in both ES and IS, and *Arc* up-regulation remained following IS, but not ES. Evidence has shown *c-Fos* and *Arc* expression is selectively upregulated in subsets of neurons across the brain related to recognition, working, and fear-related memory processes [69], and that they may also take part in synaptic plasticity circuit reorganization [70, 71]. These genes may also represent immediate early genes that are important in the general stress response, regardless of ES or IS, and change over time based on differential influences of ES and IS on the stress neurocircuit. While ES and IS displayed some similarities in gene expression in this study, the unique differential expression between ES/IS treatments across regions may help elucidate how part(s) of these inflammatory pathways change based on ES/IS influences. For example, differences in gene expression levels related to cellular repair, protein trafficking, and neurotransmission may suggest they influence how whole pathways, or portions thereof, are activated and result in either neurodestructive or neuroprotective outcomes. ES showed higher up-regulation of these immune-related pathway scores compared to IS. This difference can be seen in both HPC and mPFC, though slight differences between the two regions were observed. However, these data do not reveal the precise pathway(s), or pathway component(s), behind this gene expression, though BLA appears to be regulating these outcomes at the circuit level.

### Manipulation of amygdalar projections

#### Amygdalar inhibition

We also found that regional responses can vary based on BLA manipulation. Optogenetic inhibition of BLA suppressed almost all gene expression in HPC regardless of whether the mice were trained with ESOi or ISOi. Only four genes were upregulated after BLA inhibition: *Ago4* and *Mb21d1* were upregulated after both ESOi and ISOi and *Stx18* and *Lyn* were only upregulated after ISOi. Further work will be required to determine whether these genes are important for BLA mediation of the immune response to stress in HPC. By comparison, after BLA inhibition, mPFC showed relatively equal up- and down-regulation of genes involved in similar inflammatory-related gene expression, regardless of whether the mice experienced ESOi or ISOi. The changes to regional regulation of inflammatory-related pathway scores are most likely a reflection of this alteration in gene transcription. Thus, for the first time, we have demonstrated that inhibition of BLA glutamatergic projections to other stress-responsive regions nearly eliminates all differential effects of ES and IS on the parainflammatory response to stress.

Putatively, under “normal” conditions where reciprocal projections of each region in the circuit are functional, mPFC’s demonstrated role in the perception of control and in mediating the consequences of stress [21, 38] would suppress BLA activation during exposure to ES. This likely drives the increases in neuroprotective signaling seen within both mPFC and HPC. By comparison, the increase in inflammatory signaling in HPC and mPFC by IS exposure may arise from an absence of BLA suppression by mPFC. Inhibition of BLA projections to these regions could thus alter normal circuit activity associated with uncontrollable stress, causing the inflammatory response of IS animals to more closely resemble that of ES animals.

Optogenetic inhibition of BLA activity also could prevent reciprocal communication with HPC, potentially blocking both BLA initiation and regulation of stress response mechanisms [54–56] and the ability of BLA to respond to contextual association signals from HPC. Similarly, inhibition of BLA would block communication with mPFC, and likely also alter communication between HPC and mPFC. Therefore, signaling within HPC, and likely to mPFC, halts; resulting in the termination of active transcription in this region. Thus, the differences between ES and IS mediated by this circuitry cannot occur normally, leading to a similar regulation of inflammatory processes in both groups. This further suggests that BLA is also important for evaluating stressor controllability [65]. Minor regional differences in expression persist which may be related to their underlying functional roles in mediating systems level interactions in the overall stress response, and the presence of an underlying general stress response as discussed above.

#### Amygdalar activation

Optogenetic activation of BLA also dysregulated differences between ES and IS exposure. ESOe mice exhibited increases in inflammatory signaling in both HPC and mPFC similar to that of animals exposed to IS, whereas ISOe animals displayed a heightened parainflammatory response and further reduction of neuroprotective functions in HPC and mPFC compared to that seen in mice exposed to IS alone, thereby further enhancing the IS phenotype. The changes to regional regulation of inflammatory-related pathway scores are most likely a reflection of this alteration in gene transcription. Therefore, activation of BLA glutamatergic projections to other stress-responsive regions also nearly eliminated the differential effects of ES and IS on the stress-induced parainflammatory response, but made the ES response more like the IS response, though minor regional differences in expression still appear. It is possible that because blue light stimulation was not continuous during ST, projections from HPC and mPFC into BLA were still able to partially inhibit BLA during ES. Another possibility is that differences reflected HPC and/or mPFC trying to compensate for increased BLA activity.

### Conclusions

Our findings are congruent with other studies suggesting that stressor parameters are important in determining the inflammatory response mechanisms [48, 72–74]. For example, acute (e.g., novelty or open field) and chronic stressors (e.g., social isolation or chronic mild stress) can heighten or suppress immune function, respectively, and intense stressors (e.g., single prolonged stress) can cause immune dysregulation [74]. Differences in gene expression data during optogenetic inhibition or activation indicates in this study a role for BLA in influencing regional parainflammatory responses to different stressor parameters.

It has been suggested that problems in processing stress involve disruptions in functioning at the circuit level, rather than a localized disturbance within a given circuit node or center [64]. Optogenetic studies often use behavioral outputs and neural recordings, both locally and at the circuit level, to assess effects of activation and inhibition of selected neurons and brain regions. It has also been recognized as a useful tool for exploring the influences of specific neuronal populations on immune responses [75]. Our current results demonstrate that optogenetic manipulations within one brain region can produce significant alterations in stress-induced immune activity (“parainflammation” [39]) that can vary across nodes of the neural circuit(s) it influences.

The ES-IS paradigm we used provides a model for assessing these parainflammatory changes mechanistically. Given increasing recognition that inflammation in the brain plays a role in neurodegenerative diseases [6–11] and neuropsychiatric disorders [7, 14, 15, 17, 76], optogenetics may thus also be useful for determining how circuit level interactions between neural activity and inflammatory mechanisms become pathological. It would also lend itself to determinations of mechanisms that mediate inflammatory outcomes at the regional level, and directly at the neuro-immune interface.

There are some important limitations to this work that must be mentioned. While Nanostring® Neuroinflammatory panels are a useful tool for assessing global changes, they are not capable of determining the precise mechanisms that regulate inflammation. Therefore, other techniques (e.g., flow cytometry or PCR) should be used to further validate and elucidate Nanostring results. Potential sex differences in the regulation of regional immune responses by BLA should also be considered. However, this work provides an important proof-of-concept that has identified global changes which provides a cornerstone for future work that plans to reveal the precise mechanisms of these signaling pathway alterations, as well as further examine the temporal course of BLA regulation of stress-induced parainflammation and assess the role(s) of other regions (e.g., mPFC) in order to understand regulation of inflammatory responses at the circuit level.

## Supplementary Information

**Additional file 1: Fig. S1**: Immune-Related Pathways within HPC and mPFC Do Not Differ Greatly between CHCO and CMTO Regardless of Excitatory or Inhibitory Optogenetic Manipulation of BLA. Heatmap displaying sample’s directed global significance pathway scores for hippocampus and medial prefrontal cortex in mocked trained control with inhibitory opto and mocked trained control with excitatory opto compared to home cage with control vector. Red denotes neuroinflammatory-related pathway gene sets whose genes exhibit extensive over-expression; green denotes gene sets with extensive under-expression. Mean scores are plotted to show how they vary across treatment conditions. **Figure S2.** CMTO Induced Low-Grade Inflammatory GeneExpression Compared to CHCO, but Did Not Further Activate the Immune System Regardlessof Excitatory or Inhibitory Optogenetic Manipulation of BLA.

## Data Availability

The datasets used and/or analyzed during the current study are available from the corresponding author upon reasonable request.

## Abbreviations

AAV: Adeno associated virus
BLA: Basolateral amygdala
CD: Cluster of differentiation
CHCO: Home cage control with optogenetics control construct
CMTOe: Mock trained control with excitatory optogenetics construct
CMTOi: Mock trained control with inhibitory optogenetics construct
ES: Escapable footshock stress
ESOe: Escapable footshock stress with excitatory optogenetics construct
ESOi: Escapable footshock stress with inhibitory optogenetics construct
eYFP: Yellow fluorescent protein
HC: Home cage control
hChR2: Channelrhodopsin 2
HPA: Hypothalamic–pituitary–adrenal axis
HPC: Hippocampus
IL: Interleukin
IS: Inescapable footshock stress
ISOe: Inescapable footshock stress with excitatory optogenetics construct
ISOi: Inescapable footshock stress with inhibitory optogenetics construct
LPS: Lipopolysaccharide
mPFC: Medial prefrontal cortex
NpHR: Halorhodopsin
PFC: Prefrontal cortex
SIH: Stress-induced hypothermia
