# Supplementary material for: Stressor control and regional inflammatory responses in the brain: regulation by the basolateral amygdala
Source: J Neuroinflammation. 2023 May 27;20:128. doi: 10.1186/s12974-023-02813-x (PMC10225081; doi:10.1186/s12974-023-02813-x)
Supplement: Supplementary file 1 — Additional file 1: Fig. S1: Immune-Related Pathways within HPC and mPFC Do Not Differ Greatly between CHCO and CMTO Regardless of Excitatory or Inhibitory Optogenetic Manipulation of BLA. Heatmap displaying sample’s directed global significance pathway scores for hippocampus and medial prefrontal cortex in mocked trained control with inhibitory opto and mocked trained control with excitatory opto compared to home cage with control vector. Red denotes neuroinflammatory-related pathway gene sets whose genes exhibit extensive over-expression; green denotes gene sets with extensive under-expression. Mean scores are plotted to show how they vary across treatment conditions. Figure S2. CMTO Induced Low-Grade Inflammatory GeneExpression Compared to CHCO, but Did Not Further Activate the Immune System Regardlessof Excitatory or Inhibitory Optogenetic Manipulation of BLA. [file 12974_2023_2813_MOESM1_ESM.docx]

**B.**

**A.**

**D.**

**C.**


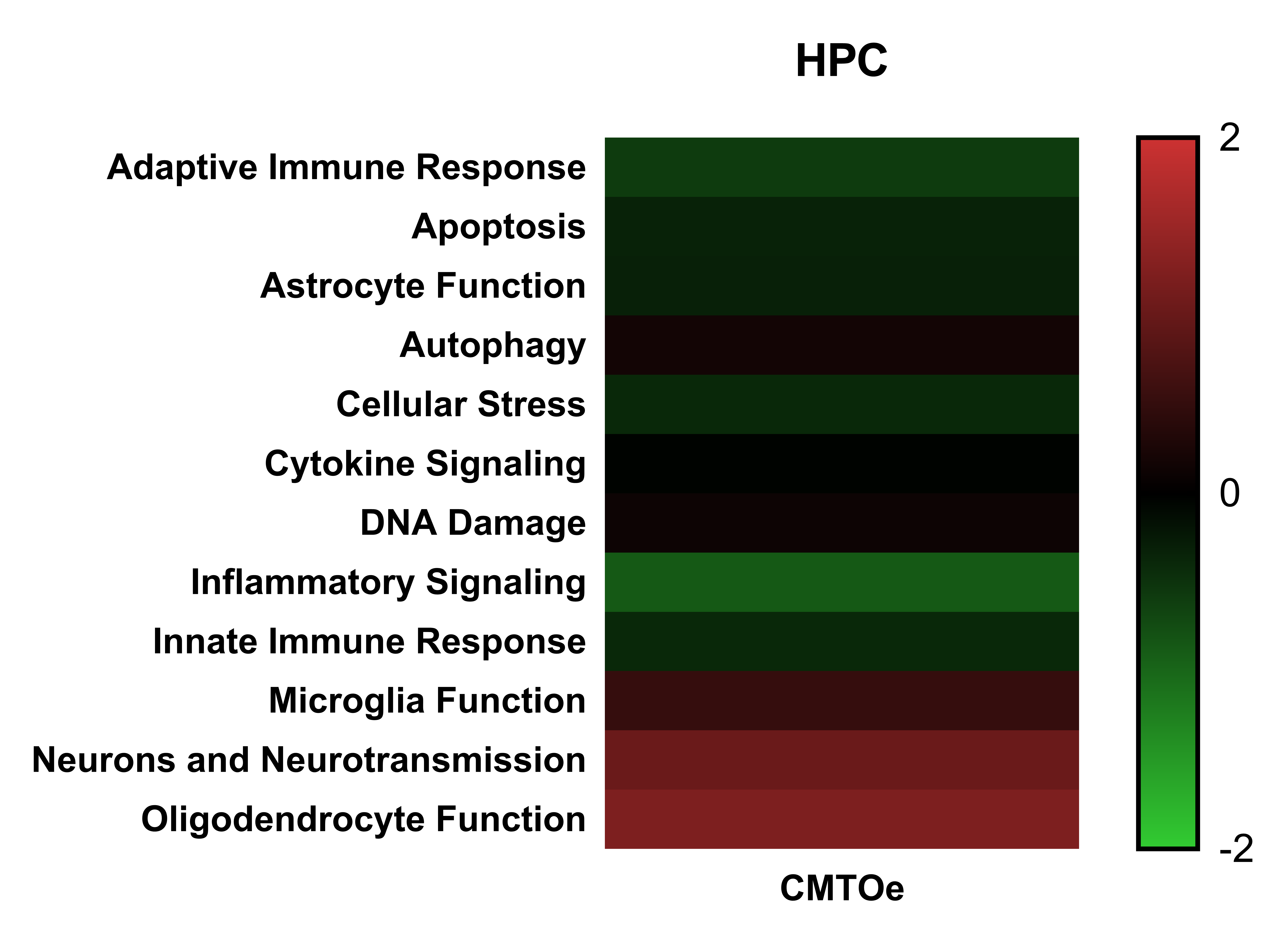

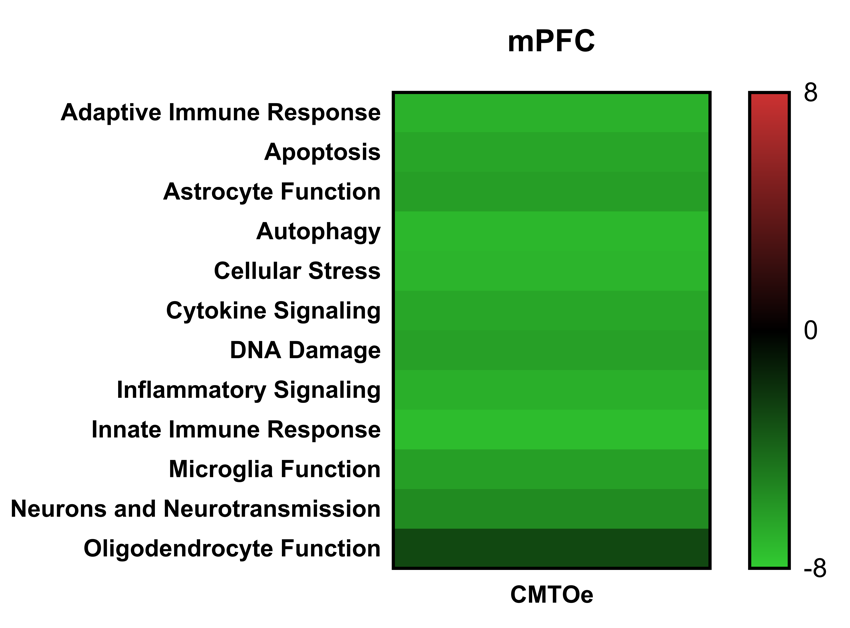

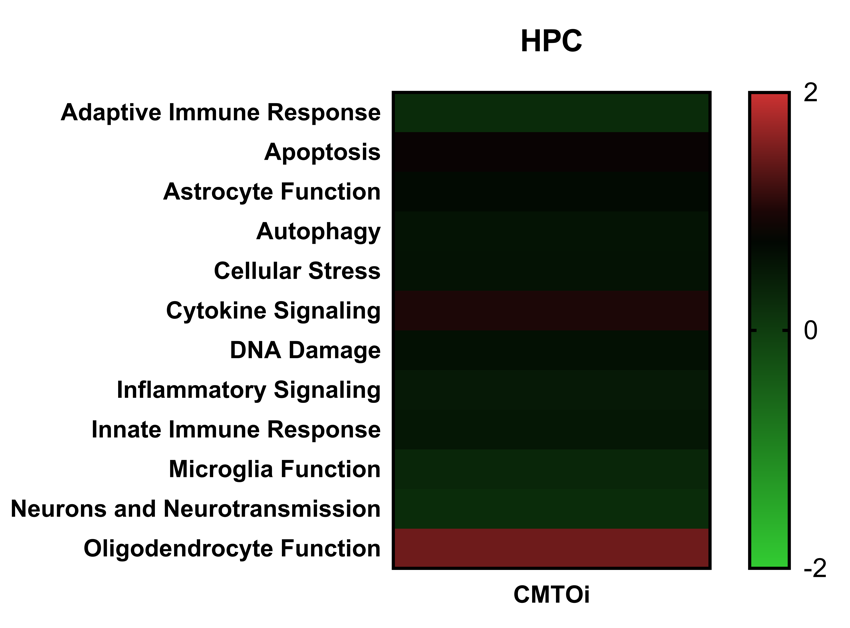

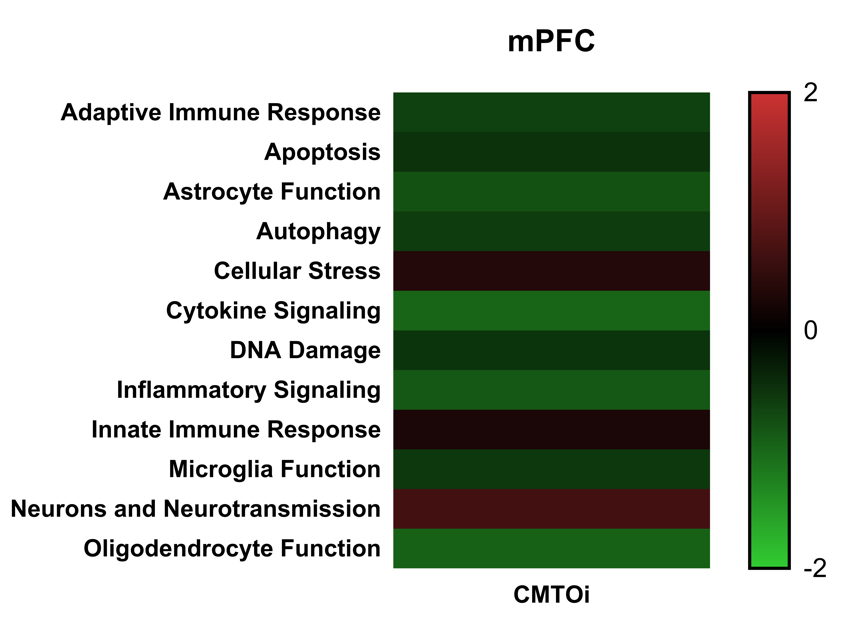


**Supplementary Figure 1: Immune-Related Pathways within HPC and mPFC Do Not Differ Greatly between CHCO and CMTO Regardless of Excitatory or Inhibitory Optogenetic Manipulation** **of BLA.** Heatmap displaying sample’s directed global significance pathway scores for hippocampus (HPC) and medial prefrontal cortex (mPFC) in (**A, B)** mocked trained control with inhibitory opto (CMTOi) and (**C, D)** mocked trained control with excitatory opto (CMTOe) compared to home cage with control vector (CHCO). Red denotes neuroinflammatory-related pathway gene sets whose genes within exhibit extensive over-expression; green denotes gene sets with extensive under-expression. Mean scores are plotted to show how they vary across treatment conditions **Color should be used for print.**

**Supplementary Figure 2: CMTO Induced Low-Grade Inflammatory Gene Expression Compared to CHCO, but Did Not Further Activate the Immune System Regardless of Excitatory or Inhibitory Optogenetic Manipulation** **of BLA.** Volcano plot displaying each gene expression levels compared to home cage (CHCO) control for **(A)** mocked trained control with inhibitory opto (CMTOi) in the hippocampus (HPC), **(B)** CMTOi in the medial prefrontal cortex (mPFC), **(C)** mocked trained control with excitatory opto (CMTOe) in HPC, and **(D)** CMTOe in mPFC. P-values were -Log10 transformed; statistically significant genes (p<0.05) fall above the horizontal line. Highly differentially expressed genes fall to either side of the zero on the x-axis. The most relevant genes are labeled in the plot.


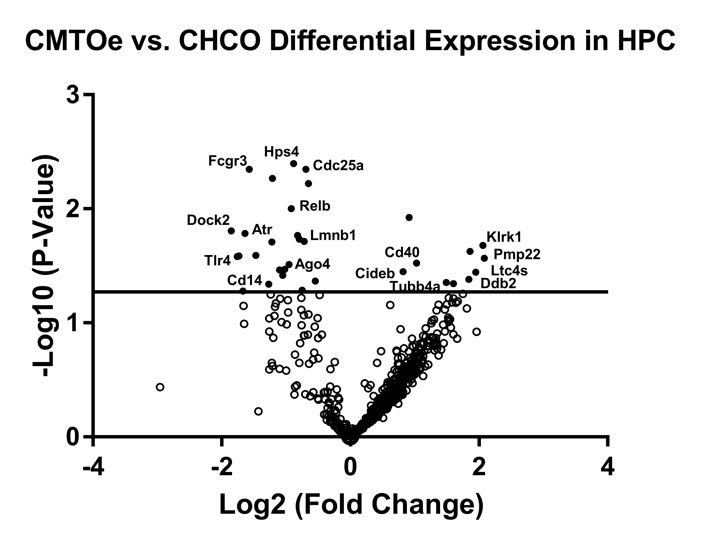

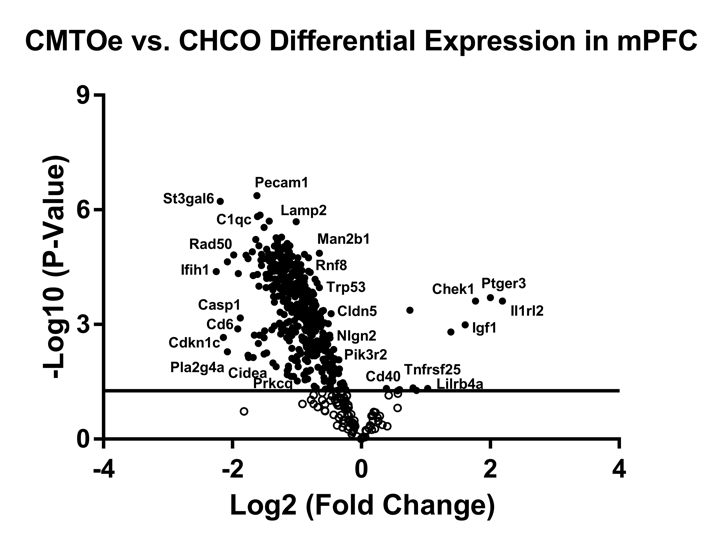

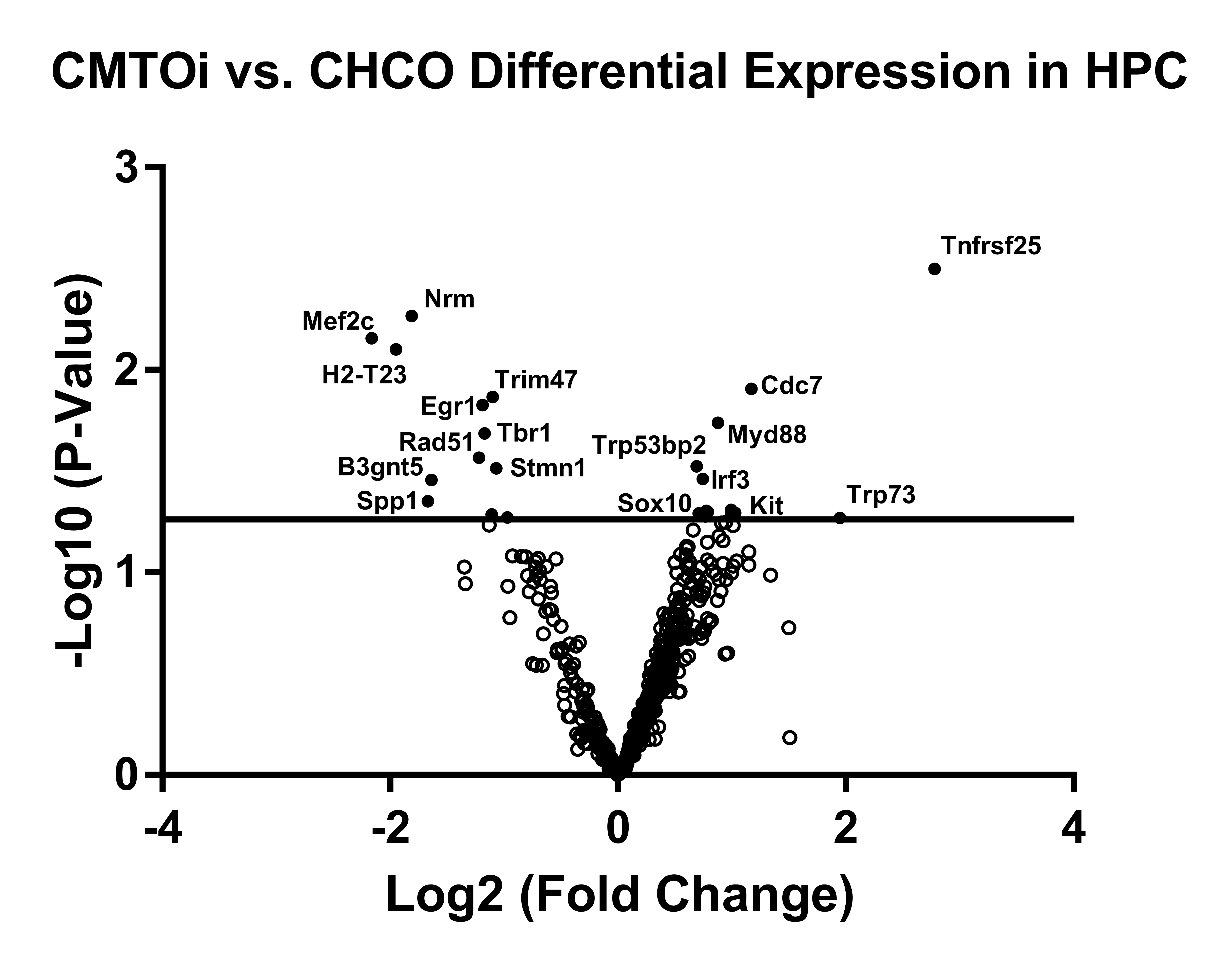

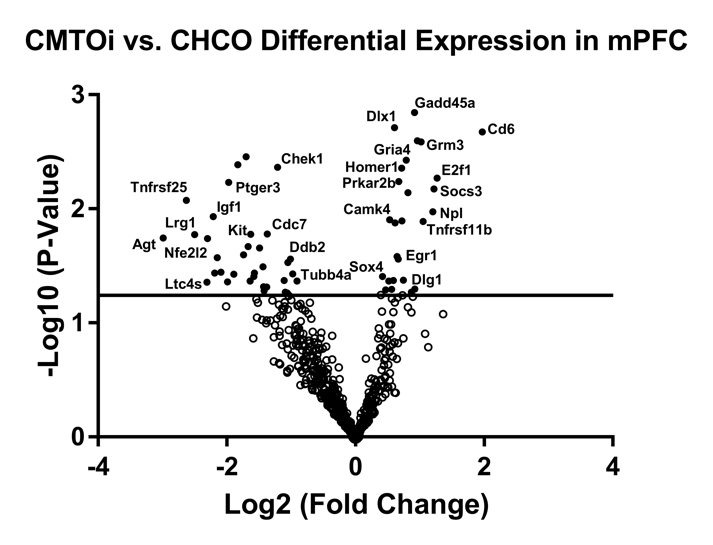


**B.**

**A.**

**D.**

**C.**
